# Bronchoalveolar lavage fluid galactomannan as a diagnostic biomarker for IPA: still a long way to go

**DOI:** 10.1186/s13054-016-1452-9

**Published:** 2016-09-19

**Authors:** Yuetian Yu, Cheng Zhu, Yuan Gao

**Affiliations:** 1 Department of Critical Care Medicine, Ren Ji Hospital, School of Medicine, Shanghai Jiao Tong University, 145, Middle Shangdong Rd, Shanghai, 200001 China; 2 Department of Emergency Medicine, Rui Jin Hospital, School of Medicine, Shanghai Jiao Tong University, Shanghai, 200025 China

In a recent issue of *Critical Care*, Maria Schroeder et al. [1] reported a prospective observational study involving 85 patients who had positive *Aspergillus* cultures or had only a positive galactomannan test. They hypothesized that a diagnostic algorithm including the detection of galactomannan in bronchoalveolar lavage fluid (BALF) would increase the diagnostic sensitivity for invasive pulmonary aspergillosis (IPA) in intensive care unit (ICU) patients. The result of this study indicated that the diagnostic sensitivity for IPA could be increased by applying a cutoff value of 0.5 in BALF galactomannan as an additional entry criterion for the AspICU clinical algorithm. Based on the knowledge of the previous research, we would like to make some remarks.

BALF galactomannan assay is recommended as an accurate marker for the diagnosis of IPA in adult and pediatric patients or even in patients receiving mold-active antifungal therapy or prophylaxis [2]. However, there is no consensus on its cutoff index and values of 0.5,1.0 and 3.0 have been reported in clinical studies. One systematic review and meta-analysis including 30 diagnostic studies evaluated the BALF galactomannan assay for diagnosing IPA and revealed that the assay had higher sensitivity with an optimal cutoff index of 1.0 compared with a cutoff index of 0.5. The sensitivity was even higher than the polymerase chain reaction (PCR) and serum galactomannan test [3]. No standardized procedure for extracting BALF has been published yet and different studies have taken different approaches to collecting BALF. As the yield of BALF is low, different methods affected the BALF assay and led to different results.

The sensitivity of galactomannan in serum varies from 29 to 100 %, especially in severely immunocompromised patients who were admitted to ICU because of infectious diseases which led to the AspICU protocol not including *Aspergillus* antigen testing [4]. It is a possible concern that factors associated with false negative results for the galactomannan assay in serum, such as prophylactic use of antifungal drugs and immunocompromised patients, can also result in false negative results when measuring galactomannan in BALF [4]. More importantly, it is difficult to identify patients who are in the early stage of IPA, particularly when the serum galactomannan test does not yield clear results and the BALF galactomannan test shows a positive result [5].

In conclusion, although the galactomannan assay in BALF is the current trend for diagnosing IPA, the cutoff index and BALF procedures need to be standardized, taking into account factors that may affect the results.

## Abbreviations

BALF, Bronchoalveolar lavage fluid; ICU, Intensive care unit; IPA, Invasive pulmonary aspergillosis; PCR, Polymerase chain reaction.
